# Unveiling SmearOFF Efficacy in Smear Layer Removal through Ultrasonic Activation Examined by Scanning Electron Microscopy

**DOI:** 10.1155/2024/8188413

**Published:** 2024-09-21

**Authors:** Hidayat Ababakr Khudhur, Diyar Khalid Bakr, Niaz Hamaghareeb Hamasaeed, Sazan Sherdl Saleem, Sohela Fakher Mahdi, Hozan Farid Tawfiq

**Affiliations:** College of Dentistry, Hawler Medical University, Erbil, Iraq

## Abstract

A layer of smear that coats the walls of root canals is produced by root canal instrumentation, which could be unfavorable to endodontic therapy. The endodontic irrigant SmearOFF is designed to effectively remove both the smear layer and bacteria concurrently. The objective of this study was to evaluate and compare the efficacy of SmearOFF and 17% EDTA in removing the smear layer across the coronal, middle, and apical thirds of root canals. Sixty-four single-canal mandibular premolar roots were chosen. Two irrigant protocols were separated into two sets of thirty-two teeth, respectively, Group 1 (6% NaOCL/SmearOFF) and Group 2 (6% NaOCl/17% EDTA.) Until X2, the ProtaperNext rotary system (Dentsply, Maillefer, Switzerland), with a COXO C-SMART Endomotor (Foshan COXO Medical instrument Co., Ltd., China) was utilized for the shaping of all teeth, the equipment settings were tuned to 300 revolutions per minute (rpm) and a torque of 3 Newton-centimeters (Ncm). Before applying the final irrigants, an initial irrigation with 6% sodium hypochlorite (NaOCl) was performed using a 27-G side-vented needle. An ultrasonic gadget, EndoUltra, was utilized to activate the irrigation. After that, the determination of how well the proposed solutions worked on the prepared teeth was conducted by scanning electron microscopy. The mean smear layer scores were lower in all three regions (coronal, middle, and apical) using 17% EDTA in comparison with the samples treated with SmearOFF. Despite that, there were no significant differences between G1, 6% NaOCL/SmearOFF and G2, 6% NaOCL/17% EDTA in smear layer removal according to Kruskal–Wallis tests and Mann–Whitney *U*-tests (*p* < 0.05). Considering the findings of this investigation, both 17% EDTA and SmearOFF serve as chelating agents, demonstrating the capability to effectively remove the smear layer. This process is facilitated with the assistance of passive ultrasonic irrigation at intervals of every third of the root canal.

## 1. Introduction

The major objectives of root canal therapy are three-dimensional cleansing, shape, as well as adequate root filling providing a sufficient seal of the root canal. Thorough debridement along with eradication of the smear layer is advantageous and may contribute to the favorable outcome of the root canal procedure [1]. Removing the smear layer is often recommended since it can include a combination of bacteria and their byproducts [2, 3]. Additionally, this layer can hinder the diffusion of intracanal medication into the dentinal tubules, so its elimination is also important for improving the adhesion of sealer cement to the canal walls [4]. This so-called smear layer has been eliminated using a variety of chemical treatments. No irrigating solution has been discovered that can effectively remove both the organic and inorganic parts of the smear layer. The sodium hypochlorite (NaOCl) and ethylenediaminetetraacetic acid (EDTA) sequential rinse method is, however, the one that is most frequently used to remove the smear layers from surfaces [1].

It has been demonstrated that EDTA has certain drawbacks, including decreased efficacy in removing the smear layer from the apical third, cytotoxicity, and a decrease in the bond strength of resin cements [5, 6]. SmearOFF (Vista Dental Inc., Racine, WI, USA), a more recent irrigating product, has been introduced. It contains surface modifiers, wetting agents, EDTA, and CHX [7]. Significant work has gone into creating irrigation techniques and/or irrigants to help with eliminating microbes from the pulpal cavity [8]. Instead of adhering to the conventional three-step approach, which typically involves initially rinsing the canal with saline to remove sodium hypochlorite (NaOCl) before introducing additional irrigants, the manufacturer suggests employing a two-step process by combining it directly with NaOCl. [9]. According to the manufacturers, it has improved calcium suspension, superior chelation, and will not precipitate when combined with NaOCl. Comparing SmearOFF to EDTA, prior research on both straight and curved root canals showed that SmearOFF was more effective in removing the smear layer [7, 10].

Yet, irrigants are often unable to reach inorganic and organic components, necessitating the employment of supplementary procedures to encourage successful removal of the smear layers [11]. Although endodontic irrigants' chelating and antibacterial properties are crucial for cleaning and sanitizing the root canal system (RCS), conventional needle irrigation may prevent these agents from reaching the dentinal tubules [12]. Furthermore, it is possible that irrigants do not consistently reach every part of the canal, particularly the apical third, due to the vapor lock effect that results from air trapping at the closed end of the root canal during irrigant delivery [13]. The antibacterial and debridement capacity of traditional syringe irrigation-transmitted solutions may be insufficient since these solutions cannot fully penetrate into the apical portions and lateral canals of the roots [14].

As a result, several tools and methods for activating irrigants have been created and suggested to raise the effectiveness and dispersion of rinses [12, 15, 16]. With the assistance of acoustic microstreaming that is conveyed at a 30 kHz ultrasonic frequency from an oscillating tool or smooth wire, passive ultrasonic irrigation (PUI) activates the irrigant solution. When compared with traditional needle irrigation, it also enhances the RCS's cleansing and disinfecting [12]. A brand-new wireless ultrasonic equipment is called EndoUltra (Vista Dental Products, Racine, WI, USA). Claims from the maker state that it is the only tool that can generate a 40 kHz frequency and successfully clean and remove the vapor locks [17]. Mobaraki and Yeşildal showed that the utilization of passive ultrasonic activation at the time of final rinse for eradicating the smear layer increases the effect of both 17% EDTA and SmearOFF solutions [18].

Although during root canal preparation the operator relies foremost on endodontic instruments, an effective irrigation protocol is of critical importance as it cleans the root canal system from the smear layer, flushes out debris, and acts as a tissue solvent, bactericidal agent, and lubricant. Investigations of smear layer removal using different irrigation protocols are important for understanding how these differences affect smear layer removal. To the best of the research team's understanding, not much is known about SmearOFF's capacity to eliminate the smear layer from canal dentinal walls. There is also a lack of sufficient studies that explore the effectiveness of the EndoUltra ultrasonic activation device with chelating agents on smear layer removal. The use of a more powerful passive ultrasonic tool with the assistance of acoustic microstreaming to remove the smear layer effectively in adjunct with SmearOFF has yet to be researched efficiently. Therefore, more research in this field is recommended, and it is a cause for concern to look into these newer irrigation protocol adjuncts to find the most updated effective treatment.

The purpose of the study was to evaluate and distinguish between the effectiveness of SmearOFF and 17% EDTA in eliminating smear layers from the apical, middle, and coronal thirds of root canals using ultrasonic activation. The null hypothesis of the study posited that the application of SmearOFF would have no effect on the removal of the smear layer from the dentinal walls of the root canals in the coronal, middle, and apical thirds.

## 2. Methods and Materials

For this investigation, sixty-four single-canal, straight, and mature mandibular premolar roots from humans were chosen. The teeth selected for the study were freshly extracted for orthodontic purposes, and the age range was limited to individuals between 15 and 25 years. This age restriction was implemented due to the acknowledged influence of tooth age on the nature of dentin and dentinal tubules. So, samples were selected depending on similar canal configuration and dentin hardness [19]. An inclusion criterion for the study encompassed teeth with straight and mature roots. Conversely, an exclusion criterion involved the exclusion of teeth showing the presence of microcracks, as determined through examination with a stereomicroscope. Additionally, radiographs of the teeth were taken buccolingually and mesio-distally to confirm the absence of any calcification, fractures, resorption abnormalities, variations in canal architecture, and endodontic treatment history. The crowns of each tooth using a periodontal scaler (Delta, Turkey), were cleaned to get rid of calculus and soft tissues.

The teeth were kept in distilled water for preservation to minimize bacterial growth and so that the surface of the dentin would not be affected by other solutions that have been shown to alter the dentin permeability and the organic and inorganic content of teeth. The teeth were used within 3 months [20]. A diamond disc (Komt, Germany) was used to decoronate the teeth 12 mm from the anatomic apex. First, a file size of size 10 K-file (Dentsply Maillefer) was used to confirm the canal patency of the samples. Following decoronation for each tooth. To simulate clinical conditions, the apices were waxed and sealed (StarWaxmodeling wax, DENTAURUM, Germany), and custom molds were created to grasp the samples using standardized cut water pipes and extremely high-consistency condensation silicone impression material (Exaplast premium putty, DETAX, Germany). The samples were distributed into two sets of thirty-two teeth depending on the kind of final rinse applied throughout instrumentation: Group 1 : 6% NaOCl/SmearOFF; Group 2 : 6% NaOCl/EDTA.

All channels were prepared using the ProTaper NEXT (PTN) rotary system (Dentsply, Maillefer, Switzerland) following the instructions given by the manufacturer using a COXO C-SMART Endomotor (Foshan COXO Medical instrument Co., Ltd., China) was used and the settings were adjusted at 300 rpm and a torque of 3 Ncm in a crown-down technique by the same operator following sequence of X1, X2. The glide path was expanded using a size 15 (Dentsply Maillefer) hand file. After using each successive ProTaper NEXT instrument, irrigation and recapitulation were conducted with a size fifteen hand file. This process involved the use of a total of 4 ml of 6% sodium hypochlorite (NaOCl), administered with a 27-G side-vented syringe needle (Stalowa Wola, Poland). The needle was positioned 2 mm from the working length (WL) and moved in a backward and forward motion [21]. Regarding irrigant solutions used in this study, NaOCl were used to dissolve the organic tissue of the smear layer at 6% concentration. This is because the tissue dissolving effect of NaOCl is related to concentration. NaOCL in higher possesses a better tissue-dissolving ability [22].

Following individual successive files, 6% NaOCl was activated for 30 seconds using EndoUltra (Vista Dental Products, USA). The root canals were then treated with either 4 ml of SmearOFF or 17% EDTA as a final rinse depending on the group to see their effect on the surface of the canal wall. The irrigants for each group were activated through the implementation of the EndoUltra procedure. (Vista Dental Products, Racine, Wisconsin, USA) for 60 seconds utilizing a sterilized stainless steel 15.02 tip at a frequency of 40,000 Hz, maintained in both upward and downward directions. As specified in the manufacturer's instructions, two millimeters away from the working length.

Thorough grooves were created on the buccal and palatal sides of the roots using diamond discs (Komt, Germany) without entering the canal space. A chisel was utilized to split the roots longitudinally. Three major divisions (coronal, middle, and apical) using tapered fissure carbide burs were made to cut two horizontal grooves perpendicular to the canal. Scanning electron microscopy (SEM) analysis was performed on one-half of each root [23, 24]. The specimens were examined using SEM under 2000x and 5000x magnification. Images were captured at the coronal, middle, and apical thirds to evaluate and compare the presence or absence of the smear layer.

A trio of calibrated evaluators assessed SEM images. The calibration process for evaluators was performed in a blind manner where each evaluator, who consisted of professional endodontic specialists, was completely unaware of the tested solutions and the purpose of the study. Each was blindly given the SEM images and was told to score the images according to a standard scoring system for smear layer without knowing which image belonged to which irrigation protocol as well as section of the root level. The inter-rater reliability assessment was performed in this manner to prevent bias. Using a standard scoring system founded on the efforts of Torabinejad et al. [25], the study considered all scores for the smear layer outlined as follows:

  SCORE 1: indicates there is no smear layer (every tubule is tidy and wide-open without the existence of a smear film on the surface of the root canal).
  SCORE 2: Moderate smear layer (the root canal surface was devoid of a smear layer; however, debris was present within the tubules).
  SCORE 3: Heavy smear layer (dentinal tubules and root canal surface were entirely coated with the smear layer).

### 2.1. Statistical Analysis

The data analysis involved utilizing IBM's SPSS software (version 26 for Windows) in New York, NY, USA. The aim was to compare smear layer scores across different canal locations (coronal, middle, and apical) by calculating mean values. Parametric statistical tests, specifically the Kruskal–Wallis test and Mann–Whitney *U*-test for multiple comparisons, were employed. The significance level was established at *p* < 0.05.

## 3. Results

What is shown in Table 1 is displayed as the mean and standard deviation of the scores attained after removing the root dentin smear layer at each section. According to the data, the mean smear layer scores were lower in all three regions (coronal, middle, and apical) using 17% EDTA in comparison with the samples treated with SmearOFF.

**Figure 1:**
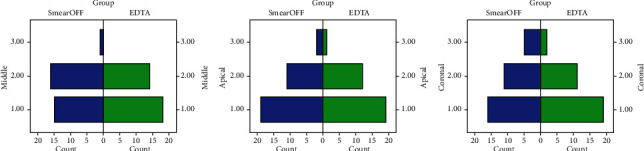
Bar graph showing the comparison of Smear layer distribution between SmearOFF and 17% EDTA at different root canal regions (coronal, middle, and apical).

The bar graphs in Figure 1 compare the scores between SmearOFF and 17% EDTA in each root canal region. It was revealed that the smear layer was successfully removed from the coronal, middle, and apical third. Clearly shown from this illustration, the scores in both irrigant groups in different root canal regions were scored as 1 and 2.

For intragroup comparisons, it was determined that there was no statistically significant difference between the groups conducted using the Kruskal–Wallis test, as shown in Table 2. The discernible distinction in the root canal areas was absent for the two irrigants evaluated in their ability to remove the smear layer from all three-thirds of the root. However, SmearOFF represents the lowest to greatest mean values in the order of apical, middle, and coronal regions. Similarly, 17% EDTA portrays the lowest mean values in the apical region, followed by the coronal and middle regions.

**Figure 2:**
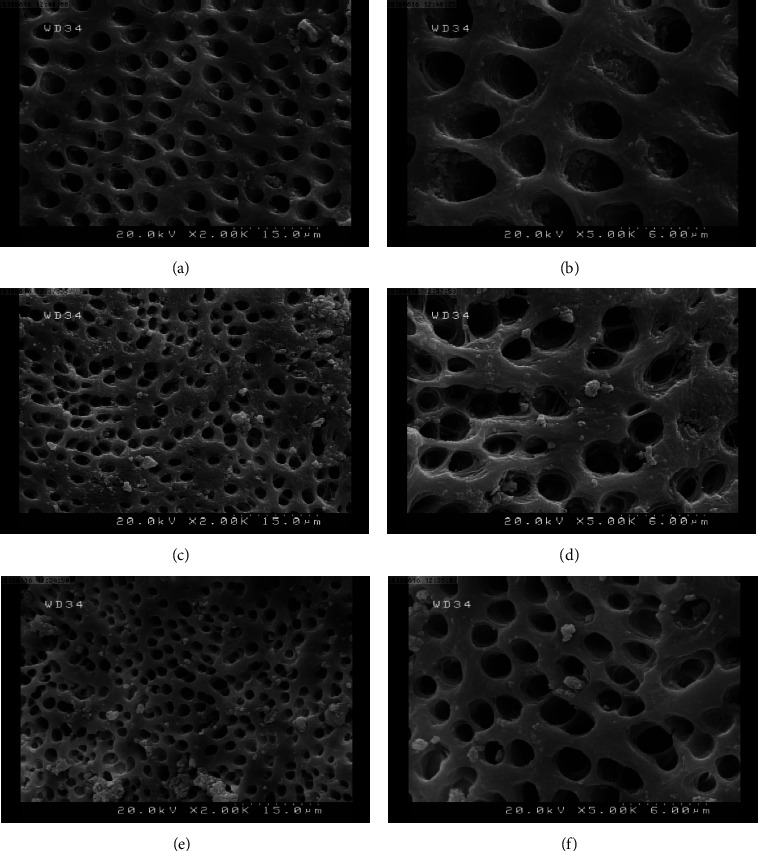
Scanning electron microscope images displaying chosen samples exposed to NaOCl/SmearOFF irrigation at the coronal (a and b), middle (c and d), and apical thirds (e and f) at magnification ×2000 and ×5000.

**Figure 3:**
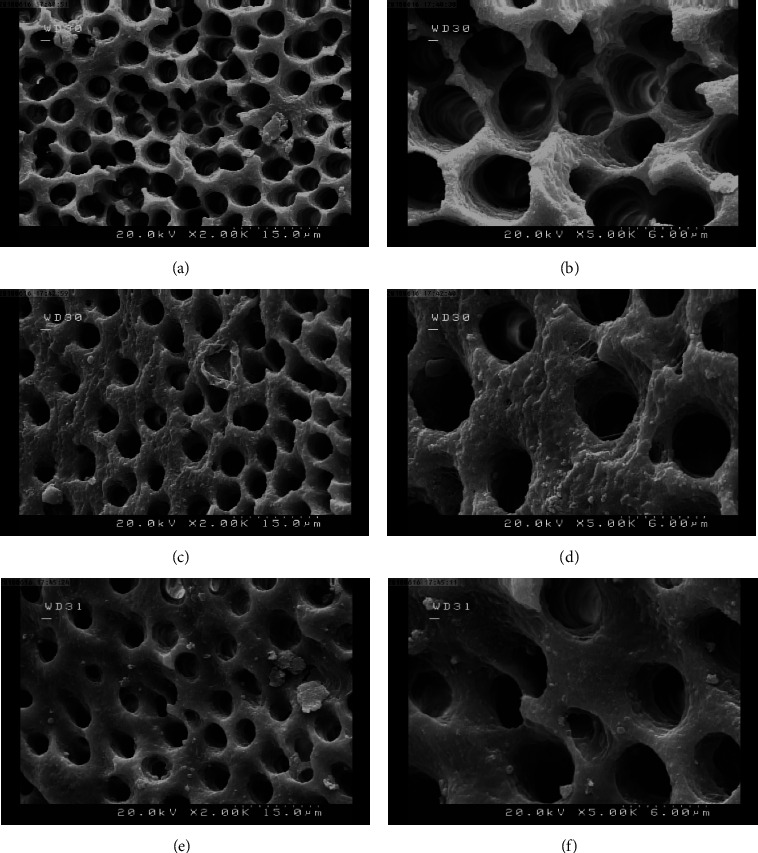
SEM images of smear layer removal by NaOCl/17% EDTA irrigation at ×2000 and ×5000. (a and b) coronal, (c and d) middle, and (e and f) apical thirds.

To determine which irrigant had the best ability to get rid of the smear layer, an intergroup comparison of the irrigants using the Mann–Whitney test was conducted (Table 3). In the present research, the difference between utilizing 17% EDTA and SmearOFF irrigating solution to eliminate the smear layer from the entire root canal dentin statistically insignificant (*p* > 0.05). Overall, 17% EDTA showed comparatively better results than SmearOFF in the removal of the smear layer at the coronal, middle, and apical regions. Figures 2 and 3 show the scanning electron microscope photographs of the removal of the smear layer between SmearOFF and 17% EDTA at various levels of the root.

## 4. Discussion

SEM images were used as part of this investigation to assess the outcomes of 17% EDTA and SmearOFF rinses with passive ultrasonic activation in removing of the smear layer. Concerning the removal, no statistically significant difference was shown between the two irrigation products and root levels. Depending on the SEM images, it was found that most of the samples from both tested solutions showed a moderate smear layer with a score of 2. In which, the root canal surface was free of smear layer, but the tubules contained some debris.

A 27-gauge side-vented needle was used to irrigate both solutions, and an EndoUltra ultrasonic device was used to activate them. Due to the tiny bore size, 27-gauge needle tips are able to penetrate the apical third more deeply [26]. The only cordless activator unit that can generate 40000 Hz frequency is EndoUltra. EndoUltra uses ultrasonic technology, which creates acoustical streaming and cavitation that enhances greater flow of movement of the irrigant and cleaning efficacy [27]. In terms of removing pulp tissue and dentin debris, passive ultrasonic irrigation is known to be better than needle irrigation. This variation can be the result of ultrasound irrigation, which enhances canal cleanliness, improves irrigant transmission to the canal system, debrides the soft tissue, and gets rid of the smear layer [28].

SmearOFF, an endodontic irrigant that is soft on the dentin and has lately been employed as a final irrigation rinse in root canal treatment, was compared to 17% EDTA in this study. Chlorhexidine, EDTA, and a surfactant agent are all included in SmearOFF [8]. Although one of the contents of SmearOFF is EDTA, a challenge associated with comparing SmearOFF and EDTA was related to ingredients because the SmearOFF contains two other ingredients do not present in 17% EDTA alone. 17% EDTA is just purely EDTA. While SmearOFF also consists of CHX and a surfactant. Because SmearOFF contains CHX, which can assist in giving antibacterial substantivity after endodontic therapy, using it may be clinically useful. Even though SmearOFF includes CHX, recent studies have demonstrated that when combined with NaOCl, SmearOFF only caused a slight color change and no precipitation could be seen [29]; nevertheless, the manufacturer asserts that SmearOFF can be used immediately following NaOCl without the necessity for saline irrigation in between [10]. The most used irrigant for removing smear layers is ethylene diamino tetra acetic acid (EDTA). At about depths of 20 to 30 m, EDTA reacts with calcium ions in dentine to cause decalcification of the dentine within 5 minutes [30]. In endodontics, EDTA is often regarded as the best chelating agent [31].

The Kruskal–Wallis test was done for comparison within groups to verify the efficacy of individual irrigants in various areas of the root canal. In all three levels of the canal surface, no significant difference existed between the groups concerning open dentinal tubule areas, implying that smear layer removal was proportional for both groups. SmearOFF and 17%EDTA's contents are nearly alike, which can clarify the outcome of these results. However, with fewer irrigating steps and an added antibacterial action of CHX, utilizing SmearOFF solution can be useful. These findings are consistent with experiments where the 17% EDTA and SmearOFF irrigation solutions did not differ significantly (*p* > 0.05) [10, 18].

Mann–Whitney test verified which irrigant is more efficacious in getting rid of the smear layer at a time where the same area of the root canal is taken into account. In this investigation, it was concluded that SmearOFF and %17 EDTA, as a final rinse in conjunction with 6% NaOCl, had comparable capability in debridement of smear layer in all three regions with no significant differences. Therefore, the current study null hypothesis was rejected. Although, in both groups, it was seen that the smear layer removal efficacy was highest in the apical region. These results were not in accordance with previous recent studies) [10, 18]. In which the apical third of all irrigation groups had less open dentin tubule regions than the middle and coronal thirds. Factors that may have caused such outcomes in other studies could be explained by the most complicated root canal form, a lack of adequate solution penetration, and increased debris penetration into the tubules utilizing the step-down technique in the apex. Additionally, one need not forget that dentinal tubule number and surface area reduce in the apical direction [25, 32, 33].

Differences in experimental design may account for why the results of this study do not agree with those of the listed prior investigations. Some altercations may be especially in relation to the anatomical features of the specimens used. However, the amount of solution and the irrigation time might also have an impact.

Because the current study was conducted in vitro as well, the findings may not necessarily support any conclusive in vivo activity of the examined drugs. The effects of the substances under inquiry in the root canal system may be influenced by blood, tissue remains, and a variety of other factors. Curved canals might present more of a challenge and make it more difficult to effectively clean the pulpal cavity. Wider canals in premolars with one root allow for deeper needle penetration; hence, outcomes in posterior teeth with narrow canals may differ. Even if there are favorable in vitro outcomes, a number of the previously described local factors in the root canal may reduce the irrigant's efficacy in vivo. One of which can result from the many chemical components present in the necrotic root canal deactivating the disinfection agent's function. In order to overcome the challenge posed by the many root canal confounding factors and enhance the connection between test results and clinical performance, a variety of ex vivo and in vivo models have been created [24, 34]. In general, both tested solutions perform better when passive ultrasonic activation is an adjunct during the final irrigation to get rid of the smear layer. Even though ultrasonic irrigation activation is successful, more research is required to develop a consistent methodology for the volume of irrigation rinse, activation time, and size of the files employed in accordance with the dimensions of the canal preparation. Additional in vitro and in vivo investigations are required in clinical management to better understand the benefits and drawbacks of SmearOFF solution. Comprehending SmearOFF solution's impact and modification on the surface of the dentin requires vital outlook into the duration of the solution in contact with the dentin [18].

## 5. Conclusion

- Passive ultrasonic irrigation, when combined with both chelating agents, 17% EDTA and SmearOFF, improved debris removal and eliminated the smear layer throughout all areas of the root canal.
- Nevertheless, the application of passive ultrasonic irrigation in conjunction with EDTA yielded the most favorable outcomes when considering factors such as the final irrigation rinse, irrigation protocol, root canal thirds, and smear layer elimination.

## Figures and Tables

**Table 1:** Descriptive statistics of the distribution of the smear layer for tested solutions at different thirds of the root.

		*N*	Mean	Std. dev.	St. error	Min.	Max.
Coronal	SmearOFF	32	1.6563	0.74528	0.13175	1.00	3.00
	EDTA	32	1.4688	0.62136	0.10984	1.00	3.00
	Total	64	1.5625	0.68718	0.08590	1.00	3.00

Middle	SmearOFF	32	1.5625	0.56440	0.09977	1.00	3.00
	EDTA	32	1.4375	0.50402	0.08910	1.00	2.00
	Total	64	1.5000	0.53452	0.06682	1.00	3.00

Apical	SmearOFF	32	1.4688	0.62136	0.10984	1.00	3.00
	EDTA	32	1.4375	0.56440	0.09977	1.00	3.00
	Total	64	1.4531	0.58905	0.07363	1.00	3.00

**Table 2:** Kruskal–Wallis test for smear layer.

Ranks					Test statistics^a,b^		
Group		Region	*N*	Mean ranks	Group		Data
SmearOFF	Data	Coronal	32	51.09	SmearOFF	Chi-square	1.115
		Middle	32	49.59		df	2
		Apical	32	44.81		Asymp. sig.	0.573
		Total	96		EDTA	Chi-square	0.021
EDTA	Data	Coronal	32	48.64		df	2
		Middle	32	48.84		Asymp. sig.	0.990
		Apical	32	48.02			
		Total	96				

^a^Kruskal–Wallis test. ^b^Grouping variable: region.

**Table 3:** Whitney *U*-test for comparison between the SmearOFF and EDTA.

Ranks				Test statistics	
Group	*N*	Mean ranks	Sum of ranks		Data

SmearOFF	32	34.95	1118.50	Mann–Whitney U	433.500
EDTA	32	30.05	961.50	Wilcoxon W	961.500
Total	64			*Z*	−1.090
				Asymp. sig. (2-tailed)	0.276

## Data Availability

The data used to support the findings of this study are available from the corresponding author upon request.
